# Fluid intake of children, adolescents and adults in Indonesia: results of the 2016 Liq.In^7^ national cross-sectional survey

**DOI:** 10.1007/s00394-018-1740-z

**Published:** 2018-06-13

**Authors:** P. W. Laksmi, C. Morin, J. Gandy, L. A. Moreno, S. A. Kavouras, H. Martinez, J. Salas-Salvadó, I. Guelinckx

**Affiliations:** 1 Geriatric Division, Department of Internal Medicine, Faculty of Medicine, Cipto Mangunkusumo Hospital, Universitas Indonesia, Jakarta, Indonesia; 2 Indonesia Hydration Working Group (IHWG), Faculty of Medicine, Universitas Indonesia, Jakarta, Indonesia; 3 Hydration and Health Department, Danone Research, Route Départemental 128, 91767 Palaiseau, France; 4 British Dietetic Association, Birmingham, UK; 5 School of Life and Medical Services, University of Hertfordshire, Hatfield, UK; 6 GENUD (Growth, Exercise, NUtrition and Development) Research Group, Faculty of Health Sciences, Instituto Agroalimentario de Aragón (IA2), Instituto de Investigación Sanitaria Aragón (IIS Aragón), Universidad de Zaragoza, Zaragoza, Spain; 7 CIBERobn (Centro de Investigación Biomédica en Red Fisiopatología de la Obesidad y Nutrición), Madrid, Spain; 8 Hydration Science Lab, University of Arkansas, Fayetteville, AR USA; 9 Division of Endocrinology, University of Arkansas for Medical Sciences, Little Rock, AR USA; 10 Hospital Infantil de México Federico Gómez, Mexico City, Mexico; 11 Human Nutrition Unit, Biochemistry and Biotechnology Department, Faculty of Medicine and Health Sciences, Hospital Universitari de Sant Joan de Reus, Institut d’Investigació Sanitària Pere Virgili, Universitat Rovira i Virgili, C/ Sant Llorenç, 21, 43201 Reus, Spain

**Keywords:** Beverages, Fluid intake, Hydration, Indonesia, Liq.In^7^, Water

## Abstract

**Purpose:**

To report daily total fluid intake (TFI) and fluid types in Indonesia according to age, sex, socio-economic status (SES) and geographic region, and compare TFI with the Indonesian adequate fluid intake (AI) recommendations.

**Methods:**

Data were collected in 32 cities over nine regions from children (4–9 years, *n* = 388), adolescents, (10–17 years, *n* = 478) and adults (18–65 years, *n* = 2778) using a fluid intake 7-day record (Liq.In^7^); socio-economic status was also recorded. The 7-day mean TFIs were compared with the AI of water set by the Ministry of Health of the Republic of Indonesia.

**Results:**

Total median fluid intakes for all age groups exceeded 2000 mL/day. At population level, TFI was associated with household income (*P* < 0.001), education (*P* < 0.001) and Indonesian geographical regions (*P* < 0.001). More than 67% of participants met the AI of water from fluids. A higher percentage of children and adolescents met the AI (78 and 80%, respectively), compared with adults (72%). Drinking water was the main contributor to TFI in all age groups (76–81%). Sugar-sweetened beverages (SSB) were consumed by 62% children, 72% adolescents and 61% of adults. An SSB intake ≥ 1 serving per day was observed for 24% children, 41% adolescents and 33% adults.

**Conclusions:**

A high percentage of the population drank enough to meet the AI of water from fluids. Water was the most frequently consumed drink; however, many participants consumed at least one serving of SSB per day. This study provides data to help direct targeted intervention programs.

**Electronic supplementary material:**

The online version of this article (10.1007/s00394-018-1740-z) contains supplementary material, which is available to authorized users.

## Introduction

Nutrition and diet surveys are used to identify public health priorities. With increasing recognition of the role of water and adequate hydration in the prevention [1, 2] and management of diseases [3, 4], it is essential that such surveys assess total water and fluid intake to inform such priorities. Age- and sex-specific recommendations on adequate intake (AI) of water have been established for many countries, for example, the USA Institute of Medicine [5]. These recommendations for total water intake (TWI) are based on the median intakes of water at population level, while the European Food Safety Authority (EFSA) [6] used data from population-level intakes, desirable volume of water per energy unit consumed, and desirable urinary osmolarity. The Indonesian Ministry of Health applied the same method as EFSA and has published dietary recommendations on the adequate intake of water (Online Resource Table S1) [7].

One of the difficulties that experts face when developing dietary reference values for water is the diversity of environmental conditions including humidity and temperature across countries and regions [8]. Daily water requirements in hot weather (40 °C) can be triple of those in cooler climates (20 °C) for any given energy expenditure [9]. For example, a recent study in Japanese adults [10] reported total water intake to be approximately 10% higher in summer than winter. Water losses and, therefore, requirements may also be influenced by other factors including humidity, air motion and clothing. Therefore, EFSA added the caveat that the AIs only apply in moderate environmental temperatures and at moderate physical activity levels [6]. In an archipelago country such as Indonesia with different temperature and humidity among major islands, it is particularly important to collect such data; however, to date there is a paucity of such information.

Recommendations are usually for adequate total water intake (TWI), which includes water from fluids and food (the latter sometimes referred to as food moisture). The water content of food varies considerably; therefore, the contribution of food to TWI will also vary. For example, it has been reported to be 19% in the USA [5], 40% in China [11] and 51% in Japan [10]. EFSA estimated in 2010 that water from food was 20-30% based on the data available at that time. However, more recent publications have estimated the contribution of food moisture to be 27% in the UK [12], 33% in the Republic of Ireland [13] and 36% in France [12]. There is a paucity of information on the contribution of water from food to TWI in hot, humid countries. When developing their recommendations for adequate intake of water the Indonesian Ministry of Health estimated that food contributed 20% to daily TWI [7]. This is in agreement with a study in Indonesia that estimated the contribution of food to TWI to be approximately 20% [14]. However, this contrasted markedly with a study from another hot, humid country, namely Bolivia that showed that food contributed an average of 50% to TWI [15]. The authors attributed this to an attempt to reduce exposure to waterborne pathogens.

Access to safe drinking water is an issue in many parts of the world [16] including, perhaps surprisingly, parts of the USA [17]. Lack of adequate safe drinking water supply is not necessarily linked to the overall wealth of the country. However, differences in individual socio-economic status (SES) have been shown to affect the risk of dehydration within a community; lower SES was associated with a higher adjusted odds ratio for elevated urine osmolality, which in turn was associated with differences in access to tap water [18]. In a rapidly developing country such as Indonesia, SES may further influence access to safe drinking water and ability to purchase bottled water.

Collecting data on total water intake has several challenges as it is difficult to obtain complete information on both food and fluid intake over a period of several days, not least due to the burden placed on participants. Fluids account for the majority of TWI and are a key target when developing interventions to increase water intake or reduce energy intake from drinks. To provide more complete data on the volume and type of fluids consumed, the present survey focused on TFI. While information is available on fluid intake from the Indonesian Total Diet Study [19], the data were collected using single 24-h recalls, which have been shown to significantly underestimate TFI [20]. In addition, these data were not analyzed according to SES.

Therefore, the primary aim of the present survey was to report daily total fluid intake (TFI) using a 7-day fluid intake (Liq.In^7^) record [21], and the contribution of different fluid types to TFI in Indonesia according to age group, sex, SES and geographic region. Earlier studies [22, 23] compared TWI in Indonesia with the EFSA recommendations [6]; since then recommendations on the AI of water have become available in Indonesia [7]. The second aim of this survey was to compare TFI with the Indonesian AIs.

## Methods

### Design and study population

The present analysis reports cross-sectional survey data for children (4–9 years), adolescents (10–17 years) and adults (18–65 years); this survey forms part of the Liq.In^7^ study. The method of recruitment, the instruments for data collection and data treatment were harmonized with the Liq.In^7^ surveys published elsewhere [24–26]. The data collection was performed in May 2016; this month was chosen for operational reasons. Temperature and humidity for the study locations were recorded using average daily information, from the following website http://www.timeanddate.com/weather/indonesia and shown in Online Resource Table S2.

Participants were randomly recruited in 32 cities over nine regions (Bali, Central Java, East Java, West Java, Yogyakarta, Jabodetabek, Sumatera, Kalimantan and Sulawesi; Online Resource Table S2) via a systematic door-to-door approach until the quotas for age, sex, habitat (urban/rural) and socioeconomic characteristics in relation to the total country population were met. Total household income, education and employment status were recorded as measures of SES.

Individuals who were not able to read and write in the language of the questionnaire (Bahasa Indonesia) or who were traveling within the next 10 days were excluded. Individuals working in the advertising, marketing, market research, the media, manufacture, distribution and/or sale of water and any kind of beverage were also excluded, as these individuals might be more aware of their fluid intake. Only one individual (regardless of age) per household was eligible to participate. If more than one member of the household was willing to take part the researcher chose the participant based on the need to satisfy the preset quotas. Pregnancy or lactation was not an exclusion criterion. There were no health-based exclusion criteria; therefore, everyone who self-reported themselves as being healthy was included.

Participants were given detailed information about the survey’s objectives, their involvement, their rights to confidentiality, risks and benefits, and a clear explanation that participation in the survey was entirely voluntary. All participants gave informed oral consent to take part in the survey. No monetary incentive was offered for taking part in the survey. All data were recorded and analyzed anonymously. The survey protocol was reviewed and approved by the University of Arkansas Review Board (ref. 14-12-376).

### Anthropometry

Height (m) and weight (kg) were measured by the researchers using suitably calibrated portable height measure and portable digital scales (GEA model number BR9202) using standard procedures [27]. The body mass index (BMI) was calculated (kg/m^2^) for adults and BMI z-score for individuals ≤ 18 years.

### Assessment of total fluid intake and the different fluid types

Participants were provided with the Liq.In^7^ record; a 7-day fluid-specific record validated for accuracy and reliability, although not in the Indonesian population [21]. The Liq.In^7^ record was presented in the official country language. A paper version of the record was delivered and explained to the participants during an interview at home. After a period of 7 days, the record was collected by the researcher after checking for completion with the participant. The Liq.In^7^ record was structured according to 12 occasions throughout the day including; awakening, meal times and periods, between meals and during the night. The participants were instructed to report all drinking events at any moment of the day with the following details: fluid type, size of the container from which the fluid was drunk, actual volume consumed, where the consumption took place and if the fluid was consumed with or without food. Food consumption was not recorded. To assist the participants in estimating the volume of fluid consumed, a photographic booklet of standard fluid containers was also provided. For children younger than 12 years, the primary caregiver was responsible for completing the record.

### Classification and analysis of fluid types

The fluids recorded were classified into the following categories: water (tap and bottled water); milk and milk derivatives; hot beverages (coffee, tea and other hot beverages); 100% fruit juices; sugar-sweetened beverages (SSB) including carbonated soft drinks (CSD); juice-based drinks; functional beverages, e.g., energy and sports drinks; ready to drink tea and coffee; flavored water; artificial/non-nutritive sweeteners beverages (A/NSB) (diet/zero/light soft drinks) and other beverages. Full details are given in Online Resource Table S3. A participant was defined as a consumer of a certain fluid type if this fluid type was consumed at least once during the 7-day period. Volumes of all categories were summed to give TFI. The proportion of individuals drinking ≤ 1 serving (250 mL) of SSB per week, 2–6 servings of SSB per week and ≥ 1 serving/day intake of SSB was calculated (Online resource Figure S3). These cut-offs were obtained from meta-analyses associating such intakes with potential risks for the development of obesity, type 2 diabetes and metabolic syndrome [28–30].

### Comparison with adequate intake of water from fluids

The observed 7-day mean TFIs were compared with the AI of water from fluids set by the Ministry of Health of the Republic of Indonesia [7] to determine the percentage of individuals in each age and sex group with TFIs meeting the AIs. The AIs for TWI were reduced by 20% to account for water in food; henceforth, this will be referred to as AI of water from fluids. Previous research has shown the contribution of food moisture to TWI to be approximately 20% [14]; the Indonesian Ministry of Health also estimated that food moisture in Indonesia was 20% when the AIs were developed [7]. The cut-offs for AI of TWI and total water from fluids can be found in Online Resource Table S1. To allow comparisons with previously published data from the Liq.In^7^ surveys, the comparison between observed intakes and the AI of water from fluids set by EFSA is also provided in the Online Resource Figure S1 [6].

### Statistical analysis

The demographic and anthropometric characteristics of the survey population are presented either as means and standard deviation for continuous variables, or numbers and percentages for dichotomous variables. Participants who did not complete the full 7-day fluid record and/or participants reporting a mean total daily fluid intake below 0.4 L/day, or higher than 6 L/day, were excluded from the analysis. No weightings were applied to the data. All intakes were skewed data (Online Resource Figure S2); therefore, TFIs are presented as medians and percentiles; mean and standard error of the mean (SEM) are given for completeness. The intakes of the different fluids are presented as median (25th − 75th percentiles) (Table 3 and Online Resource Table S4a–c). The mean and standard error of mean (SEM) of the different fluid types can be found as Online Resource Tables S5a and b. Intakes are estimated values for all participants, including non-consumers. Between-group comparisons were tested by Wilcoxon rank tests for continuous variables. All statistical tests were two tailed and as there were multiple comparisons, the significance level was set at *P* < 0.001. All analyses were performed using the SPSS software version 22.0 (SPSS Inc, Chicago, IL).

## Results

### Sample description

The demographic and anthropometric characteristics of the survey population are shown in Table 1. The mean ages of the three groups (children, adolescents and adults) were 6.4 (± 1.7), 13.4 (± 2.3) and 35.5 (± 12.1) years, respectively. The mean BMIs were 21.7 (± 14.4) kg/m^2^ for children, 22.3 (± 16.2) kg/m^2^ and 23.2 (± 5.5) kg/m^2^ for adolescents and adults respectively. Household income was similarly distributed for all age groups. The regions of Jabodetabek (an urban area of Jakarta) and Sumatera represented over 40% of the sample, while Bali and Yogyakarta represented less than 6% in all age groups.

**Table 1:** Demographic and anthropometric characteristics of the survey population (*n* = 3644), by age categories

	4–9 years	10–17 years	18–65 years
Sample size^a^	388 (11)	478 (13)	2778 (76)
Males	244 (63)	278 (58)	1256 (45)
Females	144 (37)	200 (42)	1522 (55)
Age^b^ (year)	6.4 ± 1.7	13.4 ± 2.3	35.5 ± 12.1
Weight^b^ (kg)	25.1 ± 9.4	44.0 ± 16.6	59.9 ± 15.5
Height^b^ (m)	1.1 ± 0.2	1.5 ± 0.2	1.6 ± 0.1
BMI *z* score^b,c^	1.4± 3.3	0.1 ± 1.9	
BMI^b,c^			23.2 ± 5.5
Region^a^			
Bali	9 (2)	7 (1)	72 (3)
Central Java	22 (6)	43 (9)	235 (8)
East Java	37 (10)	49 (10)	326 (12)
West Java	50 (13)	65 (14)	289 (10)
Yogyakarta	5 (1)	10 (2)	77 (3)
Jabodetabek	80 (21)	95 (20)	582 (21)
Sumatera	89 (23)	107 (22)	601 (22)
Kalimantan	52 (13)	53 (11)	335 (12)
Sulawesi	44 (11)	49 (10)	261 (9)

*BMI* body mass index

^a^Data are expressed as numbers (percentage) for categorical variables

^b^Data are presented as mean ± standard deviation for continuous variables

^c^Data are expressed in kg/m^2^ for adults and in BMI *z* score for 4–17-year-old children

### Daily total fluid intake

The daily TFIs for each sex, age group and geographic region are shown in Table 2; there were no significant differences by sex in any age group. However, there was a variation of over 1.5 L/day in fluid intakes for all age and sex categories. Total median (25th–75th percentiles) fluid intakes for the three age categories were 2156 (1430–2896) mL/day and 2080 (1436–3164) mL/day for boys and girls aged 4–9 years; 2460 (1674–3164) mL/day and 2379 (1627–3012) mL/day for males and females aged 10–17 years and 2553 (1822–3402) mL/day and 2640 (1836–3515) mL/day for adult men and women, respectively. TFI was significantly different (*P* < 0.0001) between Indonesian regions.

**Table 2:** Daily total fluid intake (mL/day) of children (4–9 years), adolescents (10–17 years) and adults (18–65 years) by sex, socioeconomic status and geographical Indonesian region

	Sex	N (%)	TFI mean ± SEM	Percentiles							*P* value^1^
				5	10	25	50	75	90	95	
Age group											
4–9 years	Total	388	2165 ± 45	874	1057	1431	2074	2880	3463	3661	
	Males	244 (63)	2169 ± 57	860	1050	1430	2059	2896	3512	3722	NS
	Females	144 (37)	2159 ± 71	865	1062	1436	2080	2870	3324	3617	
10–17 years	Total	478	2488 ± 49	877	1247	1651	2422	3138	3862	4468	
	Males	278 (58)	2499 ± 65	769	1251	1674	2460	3164	3797	4529	NS
	Females	200 (42)	2472 ± 74	881	1186	1627	2379	3012	3920	4348	
18–65 years	Total	2778	2721 ± 22	1006	1335	1827	2599	3465	4398	5004	
	Males	1256 (45)	2678 ± 33	976	1300	1822	2553	3402	4322	4849	NS
	Females	1522 (55)	2756 ± 31	1030	1350	1836	2640	3515	4459	5108	
Household income (Rupiah)^2^											
< 750,001–900,000		390 (11)	2464 ± 56	1023	1236	1638	2279^e,f^	3066	4049	4727	*P* < 0.0001
900,001–1,250,000		731 (20)	2483 ± 38	911	1189	1714	2396^e,f^	3251	3862	4263	
1,250,001–1,750,000		571 (16)	2562 ± 46	1050	1247	1707	2457^e^	3257	4059	4711	
1,750,001–2,500,000		794 (22)	2637 ± 42	971	1231	1717	2528	3358	4331	5081	
2,500,001–4,000,000		808 (22)	2802 ± 42	899	1350	1881	2746^a,b,c^	3600	4458	5013	
4,000,001–> 7,000,000		350 (10)	2830 ± 64	1082	1354	1896	2748^a,b^	3551	4544	5199	
Indonesian region											
Bali		88 (2)	2314 ± 93	951	1227	1749	2243	2880	3569	3877	*P* < 0.0001
Central Java		300 (8)	2312 ± 54	977	1192	1626	2188	2952	3518	4155	
East Java		412 (11)	2611 ± 55	739	1113	1837	2612	3311	4027	4627	
West Java		404 (11)	2168 ± 46	902	1090	1500	2022	2736	3401	3808	
Yogyakarta		92 (3)	2887 ± 124	1223	1395	2077	2779	3582	4686	5363	
Jabodetabek		757 (21)	2989 ± 42	1191	1559	2134	2899	3732	4681	5106	
Sumatera		797 (22)	2942 ± 44	960	1448	2021	2876	3729	4632	5230	
Kalimantan		440 (12)	2208 ± 45	965	1121	1492	2031	2827	3531	3849	
Sulawesi		354 (10)	2525 ± 62	974	1197	1630	2331	3267	4136	4864	

*NS* not statistically significant, *TFI* total fluid intake, *SEM* standard error of the mean

^1^Wilcoxon test to compare medians between sex and region

^2^Wilcoxon signed-rank test (*P* < 0.0001) was used to compare medians between household income

^a^Significantly different from < 750,001 to 900,000

^b^Significantly different from 900,001 to 1,250,000

^c^Significantly different from 1,250,001 to 1,750,000

^d^Significantly different from 1,750,001 to 2,500,000

^e^Significantly different from 2,500,001 to 4,000,000

^f^Significantly different from 4,000,001 to > 7,000,000

### Comparison of total fluid intakes with adequate intakes set by the Institute of Medicine, Indonesia (2014) according to age and sex categories

**Fig. 1:**
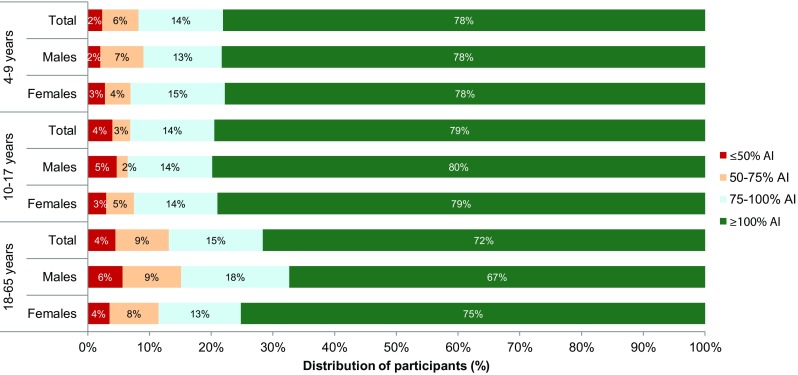
Proportion (%) of participants showing different intake levels of water from fluids compared to the age- and sex-specific adequate intake (AI) recommended by the Indonesian Ministry of Health (2012) [8]

Figure 1 shows the proportion of participants who drank more or less than the Indonesian AI of water from fluids (Online Resource Table S1) [7]. Online Resource Figure S1 shows TFI compared with the AI of water from fluids set by EFSA [6]. In all age and sex categories, at least 67% of participants met the AI of water from fluids. Table 3 shows the number and percentage of participants meeting or exceeding the recommendations according to region, household income, education level and employment status. Across the age groups, West Java had the lowest number of participants meeting or exceeding the AIs. The number of participants meeting or exceeding the AI increased with income for adults although this was less apparent in children and adolescents. Neither education level nor employment status appeared to influence the number of participants meeting or exceeding the AI. Children not meeting the AI were drinking less than 315 mL/day than the recommendations. This increased to 399 mL/day for adolescents and 531 mL/day for adults.

**Table 3:** Participants meeting or exceeding the adequate intake recommendations for water intake from fluids [7] by region, household income, education level and respondents employment status

	4–9 years		10–17 years		18–65 years	
	≥ AI (N)	≥ AI (%)	≥ AI (N)	≥ AI (%)	≥ AI (N)	≥ AI (%)
Region						
Bali	7	78%	6	86%	42	58%
Central Java	17	77%	31	72%	148	63%
East Java	26	70%	42	86%	245	75%
Jabodetabek	72	90%	86	91%	471	81%
Kalimantan	40	77%	36	68%	196	59%
Sulawesi	28	64%	41	84%	179	69%
Sumatera	81	91%	87	81%	478	80%
West Java	28	56%	44	68%	168	58%
Yogyakarta	4	80%	7	70%	63	82%
Household income						
< 750,001–900,000	22	65%	32	80%	208	66%
900,001–1,250,000	55	80%	60	70%	407	71%
1,250,001–1,750,000	49	71%	56	77%	304	71%
1,750,001–2,500,000	80	80%	92	82%	403	69%
2,500,001–4,000,000	68	83%	106	85%	452	75%
4,000,001–> 7,000,000	29	85%	34	79%	216	79%
Education level						
Primary school	49	82%	96	82%	246	69%
Junior high school	57	81%	109	75%	378	67%
Senior high school	171	78%	158	82%	1141	74%
Diploma/junior college	10	67%	4	67%	77	69%
College/university	16	70%	13	76%	148	75%
Respondent employment status^a^						
Housewife	256	80%	210	80%	807	76%
Not working/seeking employment	2	67%	10	83%	121	72%
Retired	0	–	2	100%	26	74%
Student	0	–	96	81%	183	76%
Employed	45	68%	62	75%	853	67%

*AI* Adequate intake

^a^Employment status of parent/caregiver for children aged ≤ 12 years of age

### Daily intake of different fluid types

**Fig. 2:**
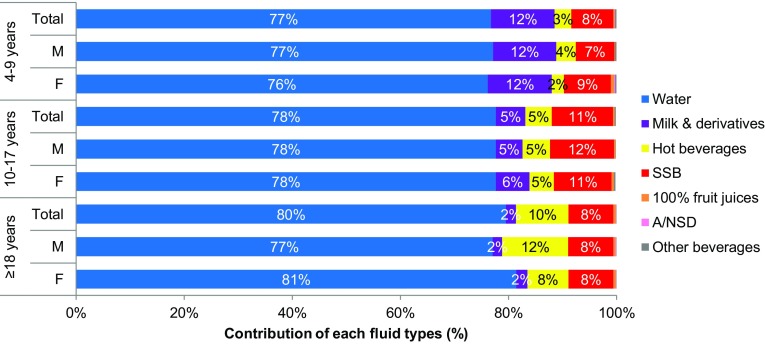
Contribution (%) to total fluid intake of the different fluid types in children (4–9 years), adolescents (10–17 years) and adults (18–65 years) by sex. *M* males; *F* females

All participants drank water, which represented 77% of TFI in children, 78% in adolescents and 80% in adults (Fig. 2). Table 4 shows the median daily intakes of the types of fluid and percentage consumers for each age group. These data were highly skewed as shown by the interquartile ranges. A higher percentage of children and adolescents drank bottled water than boiled tap water. This pattern was different in adults with an intake of 617 mL/day of boiled tap water and 480 mL/day of bottled water. The percentage of individuals consuming hot beverages increased with age (33% of children, 53% of adolescents and 73% of adults) although volumes consumed were comparatively small; tea was more frequently drunk than coffee. Consumption of milk and its derivatives decreased with increasing age. Sugar-sweetened beverages were consumed at least once a week by 62% children, 72% adolescents and 61% of adults, with ready-to-drink tea being the most frequently consumed SSB. Data according to age group are shown in Online Resource Table S4 a–c. In the total survey population, 41% of children, 29% of adolescents and 41% of adults drank ≤ 1 serving of SSB per week. An intake of 2–6 servings of SSB per week was recorded by 34% of children, 29% of adolescents and 26% of adults. A higher proportion of adolescents, children and adults consumed ≥ 1 servings of SSB per day (41, 24, and 33%, respectively) (Figure S3 in online resources).

**Table 4:** Median (P25–P75) daily intake (mL/day) of different types of fluids, and the percentage of consumers of every category, among Indonesian children (4–9 years), adolescents (10–17 years) and adults (18–65 years)

	4–9 years (*n* = 388)		10–17 years (n = 478)		18–65 years (*n* = 2778)	
	Median (P25–P75)	% consumers	Median (P25–P75)	% consumers	Median (P25–P75)	% consumers
Water	1449 (1037–2298)	100	1856 (1222–2465)	100	2006 (1371–2824)	100
Bottled water	470 (0–1663)	63	660 (0–1856)	66	480 (0–1999)	63
Boiled tap water	200 (0–1285)	53	478 (0–1562)	56	617 (0–1853)	55
Milk and derivatives	150 (0–377)	69	4 (0–214)	50	0 (0–27)	27
Hot beverages	0 (0–64)	33	31 (0–192)	53	180 (0–391)	73
Coffee	0 (0–0)	5	0 (0–0)	21	0 (0–172)	47
Tea	0 (0–48)	30	0 (0–144)	44	25 (0–211)	52
SSB	69 (0-240)	62	173 (0-412)	72	85 (0-309)	61
CSD	0 (0–0)	5	0 (0–0)	11	0 (0–0)	10
Juice-based drinks	0 (0–51)	34	0 (0–51)	33	0 (0–0)	23
Functional beverages	0 (0–0)	6	0 (0–0)	13	0 (0–0)	14
RTD tea and coffee	0 (0–130)	49	69 (0–240)	62	32 (0–214)	53
Flavored water	0 (0–0)	8	0 (0–0)	10	0 (0–0)	9
100% fruit juices	0 (0–0)	7	0 (0–0)	8	0 (0–0)	9
A/NSB	0 (0–0)	1	0 (0–0)	2	0 (0–0)	2
Other beverages	0 (0–0)	4	0 (0–0)	3	0 (0–0)	2

*SSB* sugar-sweetened beverages; *CSD* carbonated sweetened drinks; *RTD* ready to drink, *A*/*NSB* artificial/non-nutritive sweetened beverages

### Associations between type of consumed and measures of social economic status

There was a gradient in consumption of bottled or boiled tap water (Table 5) with participants in the highest income bracket consuming significantly more bottled water (*P* < 0.0001) and significantly less tap water (*P* < 0.0001). There was a low median (25–75th) intake of 34 (0–1162) mL/day of bottled water in the lowest income households. A similar gradient was observed between education level and types of water consumed with participants in the lowest education group consuming significantly more tap water (*P* < 0.0001) and significantly less bottled water than participants in the two most educated groups (*P* < 0.0001) (Table 6). No relationship was observed between type of water consumed and employment status (Table S7 in Online Resources).

**Table 5:** Daily intake (mL/day) of tap water and bottled water according to household income in the Indonesian population

Household income [rupiah (Rp)]	Mean	± SEM	Percentiles						
			P5	P10	P25	P50	P75	P90	P95
	Bottled water								
< 750,001–900,000	648	± 51	0	0	0	34^d,e,f^	1162	2188	2880
900,001–1,250,000	711	± 38	0	0	0	57^d,e,f^	1254	2366	2890
1,250,001–1,750,000	770	± 49	0	0	0	63^e,f^	1299	2514	3416
1,750,001–2,500,000	986	± 43	0	0	0	264^a,b,e,f^	1822	2841	3410
2,500,001–4,000,000	1595	± 47	0	0	210	1544^a,b,c,d^	2503	3497	4030
4,000,001–> 7,000,000	1846	± 72	0	0	824	1832^a,b,c,d^	2706	3685	4434
	Tap water								
< 750,001–900,000	1259	± 60	0	0	0	1139^e,f^	1884	2818	3497
900,001–1,250,000	1239	± 43	0	0	0	1175^e,f^	2023	2835	3362
1,250,001–1,750,000	1285	± 51	0	0	0	1200^e,f^	2160	2913	3381
1,750,001–2,500,000	1092	± 45	0	0	0	807^e,f^	1870	2816	3586
2,500,001–4,000,000	616	± 38	0	0	0	0^a,b,c,d^	1097	2298	3028
4,000,001–> 7,000,000	440	± 50	0	0	0	0^a,b,c,d^	14	2044	2686

Wilcoxon signed-rank test (*p* < 0.0001) was used to compare medians of tap water or bottled water between household income

^a^Significantly different from < 750,001 to 900,000

^b^Significantly different from 900,001 to 1,250,000

^c^Significantly different from 1,250,001 to 1,750,000

^d^Significantly different from 1,750,001 to 2,500,000

^e^Significantly different from 2,500,001 to 4,000,000

^f^Significantly different from 4,000,001 to > 7,000,000

**Table 6:** Daily intake (mL/day) of total fluid intake, tap water and bottled water according to education level in the Indonesian population

Education level	Mean	±SEM	Percentiles						
			P5	P10	P25	P50	P75	P90	P95
	Bottled water								
Primary school (*n* = 531)	769	± 49	0	0	0	71^c,d,e^	1371	2393	3110
Junior high school (*n* = 783)	868	± 41	0	0	0	103^c,d,e^	1646	2640	3262
Senior high school (*n* = 1961)	1187	± 29	0	0	0	793^a,b^	2107	3094	3762
Diploma/junior college (*n* = 132)	1479	± 119	0	0	59	1296^a,b^	2434	3645	4288
College/university (*n* = 237)	1348	± 89	0	0	0	1076^a,b^	2170	3320	3893
	Tap water								
Primary school (*n* = 531)	1290	± 55	0	0	0	1157^c,d,e^	2065	2997	3723
Junior high school (*n* = 783)	1137	± 43	0	0	0	982^c,d^	1929	2822	3557
Senior high school (*n* = 1961)	910	± 26	0	0	0	43^a,b^	1696	2620	3153
Diploma/junior college (*n* = 132)	653	± 90	0	0	0	0^a,b^	1331	2331	2755
College/university (*n* = 237)	859	± 78	0	0	0	0^a^	1549	2833	3306

Wilcoxon signed-rank test (*p* < 0.0001) was used to compare median of total fluid intake, bottled water or tap water between education level

^a^Significantly different from primary school

^b^Significantly different from junior high school

^c^Significantly different from senior high school

^d^Significantly different from diploma/junior college

^e^Significantly different from college/university

## Discussion

This cross-sectional survey reports TFI and fluid type for children, adolescents and adults aged 4–65 years in Indonesia in an attempt to study the fluid intake pattern across nine regions of Indonesia. In the present survey, 67–80% of participants reported daily TFIs that met the Indonesian recommendations on adequate intake [7]. These rates are higher than previous data from Indonesia 55–70% [22, 23] where EFSA recommendations were used [6]. While undoubtedly the use of Indonesian recommendations is more appropriate and may account for the differences reported, there are other possible explanations. For example, recently there has been an increased emphasis in promoting the drinking of water in Indonesia. Water, as opposed to fluids generally, is now included in Indonesia’s food-based dietary guidelines Tumpeng Gizi Seimbang (TGS, Balanced Nutritional Pyramid) [31]. While this, and other, campaigns may have increased public knowledge and awareness of healthy hydration, to date there are no published evaluations of the impact these initiatives may have had. Improved access to drinking water may also have been a factor in this apparent increase in participants meeting the recommended AI for water from fluids. However, improving access to water for drinking and sanitation is a health priority in Indonesia [32] and access to, at least, basic drinking water has increased from 70% of the population in 1990 [33] to nearly 80% in 2015 [16]. Despite this, there are still disparities in access due to geographical and socioeconomic differences [34]. However, it is also important to consider the representativeness of the present study as this increase may be due to the voluntary nature of participation, which, by default, leads to a select group of participants who may have been better motivated to consume higher volumes.

Data from another Indonesian study conducted, under varying climatic conditions, showed intakes for adolescents and adults similar to the present study [35]. However, it should be noted that the areas surveyed in both studies although overlapping are geographically and, therefore, climatically different. In addition to the differences in sample size, the age ranges were also different (10–17 vs. 15–18 years and 18–65 vs. 19–55 years); younger children were not included in the earlier study. Another cross-sectional study of urban school children reported similar intakes to the present study [14]. The study by Hardinsyah et al. used a 7-day recall (semi-quantitative food frequency questionnaire) [35], and the second study 2-day, 24-h recalls [14]; neither methodology was validated for assessing TFI. The more recent Indonesian Total Diet Study [19] reported an average TFI of 1317 mL/day, with adults having an average intake of nearly 1500 mL/day. It is interesting to note that despite the volumes reported in the aforementioned study, nearly half of the population was described as dehydrated based on urine specific gravity and symptoms of dehydration [19]. Secondary analysis of these data found that the dehydration risk was related to ecological (geographical) area and fluid intake [36]. The present survey also showed that geographic region was significantly associated with TFI. As hydration status was not assessed in the present study, no association between the TFI and dehydration risk could be assessed. However, it is important to note that while the majority of participants in the present study met, or exceed the recommended AIs, those not drinking enough to meet these recommendations would need to drink between 351 and 531 mL/day (at least one to two servings) depending on age. Given the association between ambient temperature and urine specific gravity [37], it is vital that more research is conducted to further elucidate the effect of climate, including ambient temperature and altitude on TFI and the risk of dehydration, especially in those not meeting AI recommendations.

Socioeconomic status may influence several factors associated with fluid intake including access to safe tap water, ability to purchase bottled water and other drinks, access to heating facilities to boil water for drinking and air conditioning. In the present survey, TFI was related to household income and education, not employment status. SES has been associated with risk of dehydration and access to clean tap water in American countries [18, 38]. Several studies have also shown socio-economic differences in patterns of different types of fluid intake, with lower SES being associated with higher consumption of sugar-sweetened beverages [39–41]. Unfortunately, fluid type was not analyzed in relation to any measure of SES in the present survey as small numbers in some fluid type groups meant that further analysis would have been meaningless. It is important to recognize that the interpretation of the influence of SES on TWI and the types of fluid consumed is confounded by factors that may or may not be included in the definition of SES. For example, a recent analysis has shown that TWI was affected by education level, ethnicity and place of birth [42].

Water was drunk by all the participants in the present survey. Earlier studies in Indonesia have also shown that water was the largest contributor to TFI [14, 35]. Intake of SSBs was low in the present survey compared with previous Indonesian studies [43, 44] although 24% of children, 41% of adolescents and 33% of adults reported consuming at least one serving per day. The intake of this amount of SSB has been associated with a 25% increased future risk of type 2 diabetes [45]. In addition, obesity is a significant risk factor for type 2 diabetes; the consumption of SSB was also independently and positively associated with and increased risk of obesity [46–48] and cardiovascular disease [28, 49]. Because of the high risk of overweight and obesity in Indonesia [50–52] and a predicted rise in the prevalence of diabetes [52] there has been a call for policies to address the increasing prevalence of type 2 diabetes [53] and the amount of sugar in SSB [54, 55]. However, a recent review has identified the need for a nationwide nutrition survey in Indonesia using appropriate methodology, before developing such policies [56]. The present study may, therefore, be useful at a national level to develop health initiatives in Indonesia in the future.

This study has several strengths; perhaps the most significant being the use of a standardized methodology that has been validated, although not in Indonesia, for assessing TFI against total body water measured by deuterium dilution [21]. The sample size was particularly large and included participants from most of the Indonesian islands. However, it is important to acknowledge possible limitations. As with all dietary surveys, the sample may have been biased towards those people most interested in this topic and/or in their diet and health and those willing to complete such research. In addition, the present sample may not be truly representative of the total population; the eastern part of Indonesia was not represented, and the sample size in some regions was small. Moreover, compared with the 2011 statistics of the Indonesian Central Bureau of Statistics (BPS), the survey sample contained fewer individuals with a lower SES and living in rural areas than the national population. In addition, these data were collected using survey procedures and as such not weighted; therefore, its representativeness of the country’s population may be questioned. The levels of overweight and obesity for adults were comparable with published figures [53] although higher than WHO figures for children [57]. Conversely, the levels of underweight for children in the present survey were substantially lower than has previously been published [55, 57]. Again, this may be a reflection of underrepresentation in lower SES groups and in rural areas. Physical activity level and occupation will greatly influence fluid intake; however, activity was not recorded in this survey. Due to the methodology with parents/carers completing the record for children under 12 years old, there is a greater potential for intakes to be under- or overestimated. No information was collected about food consumption during the study period and, therefore, it was not possible to estimate the contribution of food moisture to TWI for this population sample. While food moisture was estimated to be 20% of TWI in the current analysis, more research is needed to confirm or refute this assumption. It should also be acknowledged that it is not possible to draw any conclusions about the hydration status of the participants as no biomarkers of hydration were measured. The present survey was conducted in May and intakes may not be representative of consumption at other times of the year especially in such a hot, humid country.

## Conclusions

The present study presents data on volume and type of fluids consumed over a 7-day period in a large sample of the Indonesian population aged 4–65 years. The majority of the survey population drank enough to meet or exceed the Indonesian recommendations. However, in those not meeting the recommendations, an extra 351–531 mL/day (1–2 average servings) would need to be consumed to reach the recommended intakes. The most frequently consumed drink was water in all age and sex categories, although a significant percentage of individuals consumed at least one serving of SSB per day, a level of consumption that has been associated in different studies with an increased risk of type 2 diabetes and obesity. With rapidly increasing levels of type 2 diabetes in Indonesia, this study highlights a possible contributing factor and suggests possible targets for intervention.

## Electronic supplementary material

Below is the link to the electronic supplementary material.

Supplementary material 1 (DOCX 909 KB)
