# Supplementary material for: Fluid intake of children, adolescents and adults in Indonesia: results of the 2016 Liq.In7 national cross-sectional survey
Source: Eur J Nutr. 2018 Jun 13;57(Suppl 3):89–100. doi: 10.1007/s00394-018-1740-z (PMC6008347; doi:10.1007/s00394-018-1740-z)
Supplement: Supplementary file 1 — Supplementary material 1 (DOCX 909 KB) [file 394_2018_1740_MOESM1_ESM.docx]

**ONLINE RESOURCES**

**FLUID INTAKE OF CHILDREN, ADOLESCENTS AND ADULTS IN INDONESIA: RESULTS OF THE 2016 LIQ.IN^7^ NATIONAL CROSS-SECTIONAL SURVEY**

*Laksmi PW,*^1,2^ *Morin C,^3^ Gandy J,^4,5^ Moreno LA,^6,7^ Kavouras SA,^8,9^ Martinez H,^10^ Salas-Salvadó J,^7,11^ Guelinckx I^3*^*

^1^ Geriatric Division, Department of Internal Medicine, Faculty of Medicine, Universitas Indonesia/ Cipto Mangunkusumo Hospital, Jakarta, Indonesia

^2^ Indonesia Hydration Working Group (IHWG), Faculty of Medicine, Universitas Indonesia, Jakarta, Indonesia

^3^ Hydration & Health Department, Danone Research, Palaiseau, France

^4^ British Dietetic Association, Birmingham, UK

^5^ School of Life and Medical services, University of Hertfordshire, Hatfield, UK.

^6^ GENUD (Growth, Exercise, NUtrition and Development) Research Group, Faculty of Health Sciences, Universidad de Zaragoza, Instituto Agroalimentario de Aragón (IA2), Instituto de Investigación Sanitaria Aragón (IIS Aragón)

^7^ CIBERobn (Centro de Investigación Biomédica en Red Fisiopatología de la Obesidad y Nutrición) Zaragoza, Spain.

^8^ Hydration Science Lab, University of Arkansas, Fayetteville, AR, USA.

^9^ Division of Endocrinology, University of Arkansas for Medical Sciences, Little Rock, AR, USA

^10^ Hospital Infantil de México Federico Gómez, México City, México.

^11^ Human Nutrition Unit, Hospital Universitari de Sant Joan de Reus, Faculty of Medicine and Health Sciences, Institut d’Investigació Sanitària Pere Virgili, Biochemistry and Biotechnology Department, Universitat Rovira i Virgili. C/ Sant Llorenç, 21, 43201 Reus (Spain).

*** Corresponding author:** Isabelle GUELINCKX, Hydration and Health department, Danone Research, Route Départemental 128, 91767 Palaiseau, France; isabelle.guelinckx@danone.com

**Table S1** Adequate intake for total water intake (water from food and fluids) and total fluid intake (sum of drinking water and all other fluids), set by the Ministry of Health of the Republic of Indonesia [7]

| **Age Group** | **Total water intake** (mL) | **Total fluid intake*** (mL) |
| --- | --- | --- |
| **Infant/Child** |  |  |
| 4-6 y | 1500 | 1200 |
| 7-9 y | 1900 | 1520 |
| **Males** |  |  |
| 10-12 y | 1800 | 1440 |
| 13-15 y | 2000 | 1600 |
| 16-18 y | 2200 | 1760 |
| 19-29 y | 2500 | 2000 |
| 30-49 y | 2600 | 2080 |
| 50-64 y | 2600 | 2080 |
| 65-80 y | 1900 | 1520 |
| **Females** |  |  |
| 10-12 y | 1800 | 1440 |
| 13-15 y | 2000 | 1600 |
| 16-18 y | 2100 | 1680 |
| 19-29 y | 2300 | 1840 |
| 30-49 y | 2300 | 1840 |
| 50-64 y | 2300 | 1840 |
| 65-80 y | 1600 | 1280 |
| **Pregnancy (+ / add)** |  |  |
| Trimester 1 | 300 | 240 |
| Trimester 2 | 300 | 240 |
| Trimester 3 | 300 | 240 |
| **Lactation (+ / add)** |  |  |
| 0-6 months | 800 | 640 |
| 7-12 months | 650 | 520 |

*y* years

**Table S2** Climatic conditions of the different Indonesian regions during the period of the survey

|  |  | **Climatic conditions** | |
| --- | --- | --- | --- |
| **Region** | **Cities** | Average Temperature* (°C) | Average Humidity* (%) |
| Bali | Kota Denpasar | 29 | 81 |
| Central Java | Koto Semarang, Kota Surakarta, Semarang | 29 | 73 |
| East Java | Koto Malang, Kota Surabaya, Malang | 30 | 78 |
| West Java | Bandung, Kota Bandung, Kota Tasikmalaya | 25 | 85 |
| Yogyakarta | Kota Yogyakarta | 29 | 76 |
| Jabodetabek | Jakarta, Kota Bekasi, Kota Bogor, Kota Depok, Kota Tangerang | 29 | 77 |
| Sumatera | Deli Serdan, Kabupaten Ogan Komering Llir, Kampar, Kota Dumai, Kota Lubuklinggau, Kota Medan, Kota Palembang, Kota Pekanbaru, Kota Pekanbaru, Kota Pematang Siantar, | 28 | 84 |
| Kalimantan | Banjar, Kota Benjarmasin, Kota Pontianak, Kota Samarinda | 28 | 85 |
| Sulawesi | Gowa, Kota Makassar, Kota Manado | 29 | 77 |

* Temperature and humidity were recorded using average daily information from [www.timeanddate.com/weather/indonesia](http://www.timeanddate.com/weather/indonesia) web site.

**Table S3** Classification of the fluid types

| Classification of fluids | Detailed Fluid types |
| --- | --- |
| **Water** |  |
| *Bottled water* | Still water bottled, still water jug/gallon, sparkling plain water bottled |
| *Boiled tap water* | Tap water filtered, boiled tap water, other water (e.g., from rain, well, river, lake) |
| **Milk & derivatives** | Hot/cold raw milk, UHT milk, powder milk, milk with flavor/powder (e.g., strawberry, vanilla, chocolate), sweetened condensed milk, yogurt milk drink, fruit shake with milk, soja/almond/rice milk |
| **Hot beverages** |  |
| *Coffee* | Coffee, coffee from coffee maker (e.g.: homemade coffee, dolce gusto, others), powder coffee, instant coffee, vending machine coffee, restaurant/franchise coffee, |
| *Tea* | homemade hot/cold tea (from tea bags), Infusions (herbal), traditional herbal tea |
| **SSB** |  |
| *Carbonated sweet beverages* (*CSD)* | Cola regular, fruit flavored sparkling regular, other carbonated soft drink, sparkling lemonade |
| *Juice-based drinks* | Fruit drink regular, juicy water, still lemonade, nectar, smoothies homemade or ready to drink, syrup/concentrated/powder juice with water |
| *Functional beverages* | Sports drink, protein drinks, tonic regular, energy drinks, flavored water enriched with vitamin/minerals, liquid or powder isotonics |
| *RTD Tea & Coffee* | Ice coffee, ice tea homemade or regular, bubble tea |
| *Flavored water* | Clear flavored water, flavored water, Infused / herbal / vegetal water |
| **100% fruit juices** | Bottled 100% fruit juice & vegetables juice, homemade freshly squeezed juice, freshly squeezed juice "take from outside", |
| **A/NSB** | Cola light/zero, fruit flavored sparkling light, fruit drink light/low in calories, ice tea light |
| **Other beverages** | Beverages identified by participant as “other than listed above”, meal replacement |

**Figure S1** Proportion (%) of participants drinking more or less than the adequate intake (AI) of water from fluids set by European Food Safety Agency (2010) [6] by age group and sex

**Figure S2** Distribution of daily total fluid intake (mL/day), over 7-day of children (4-9 years), adolescents (10-17 years) and adults (18-65 years)

**Table S4a** Median (P25-P75) daily intake (mL/day) of different fluid types and the percentage of consumers among Indonesian children (4-9 years) by gender

|  | **4-9 years (n=388)** | | | |
| --- | --- | --- | --- | --- |
|  | Males (n=244) | | Females (n=144) | |
|  | Median (P25-P75) | % consumers | Median (P25-P75) | % consumers |
| **Total Fluid Intake** | **2059 (1430- 2896)** | **100** | **2080 (1436-2870)** | **100** |
| **Water** | 1433 (1010-2360) | 100 | 1525 (1068-2211) | 100 |
| *Bottled water* | *361 (0-1498)* | *59* | *757 (0-1771)* | *68* |
| *Boiled tap water* | *527 (0-1378)* | *56* | *0 (0-1211)* | *49* |
| **Milk & derivatives** | 160 (0-377) | 70 | 137 (0-377) | 67 |
| **Hot beverages** | 0 (0-74) | 35 | 0 (0-42) | 29 |
| *Coffee* | *0 (0-0)* | *5* | *0 (0-0)* | *4* |
| *Tea* | *0 (0-68)* | *32* | *0 (0-15)* | *26* |
| **SSB** | 63 (0-221) | 59 | 109 (0-260) | 67 |
| *CSD* | *0 (0-0)* | *5* | *0 (0-0)* | *6* |
| *Juice-based drinks* | *0 (0-50)* | *32* | *0 (0-57)* | *37* |
| *Functional beverages* | *0 (0-0)* | *5* | *0 (0-0)* | *6* |
| *RTD Tea & Coffee* | *0 (0-118)* | *47* | *27 (0-147)* | *52* |
| *Flavored water* | *0 (0-0)* | *7* | *0 (0-0)* | *10* |
| **100% fruit juices** | 0 (0-0) | 5 | 0 (0-0) | 10 |
| **A/NSB** | 0 (0-0) | 0 | 0 (0-0) | 3 |
| **Other beverages** | 0 (0-0) | 3 | 0 (0-0) | 6 |

*SSB* sugar sweetened beverages, *CSD* carbonated sweetened drinks, *RTD* ready to drink, *A/NSB a*rtificial/non-nutritive sweetened beverages

**Table S4b** Median (P25-P75) daily intake (mL/day) of different fluid types and the percentage of consumers among Indonesian adolescents (10-17 years) by gender

|  | **10-17 years (n=478)** | | | |
| --- | --- | --- | --- | --- |
|  | Males (n=278) | | Females (n=200) | |
|  | Median (P25-P75) | % consumers | Median (P25-P75) | % consumers |
| **Total Fluid Intake** | **2460 (1674-3164)** | **100** | **2379 (1627-3012)** | **100** |
| **Water** | 1847 (1251-2465) | 100 | 1879 (1200-2464) | 100 |
| *Bottled water* | 777 (0-1849) | 68 | 346 (0-1969) | 63 |
| *Boiled tap water* | 341 (0-1604) | 54 | 777 (0-1529) | 58 |
| **Milk & derivatives** | 0 (0-184) | 49 | 29 (0-239) | 52 |
| **Hot beverages** | 34 (0-229) | 55 | 2 (0-163) | 50 |
| *Coffee* | 0 (0-0) | 23 | 0 (0-0) | 19 |
| *Tea* | 0 (0-167) | 45 | 0 (0-125) | 43 |
| **SSB** | 183 (0-436) | 71 | 171 (0-371) | 74 |
| *CSD* | 0 (0-0) | 11 | 0 (0-0) | 12 |
| *Juice-based drinks* | 0 (0-51) | 33 | 0 (0-51) | 34 |
| *Functional beverages* | 0 (0-0) | 12 | 0 (0-0) | 16 |
| *RTD Tea & Coffee* | 76 (0-283) | 63 | 57 (0-205) | 61 |
| *Flavored water* | 0 (0-0) | 7 | 0 (0-0) | 13 |
| **100% fruit juices** | 0 (0-0) | 7 | 0 (0-0) | 10 |
| **A/NSB** | 0 (0-0) | 2 | 0 (0-0) | 3 |
| **Other beverages** | 0 (0-0) | 1 | 0 (0-0) | 6 |

*SSB* sugar sweetened beverages, *CSD* carbonated sweetened drinks, *RTD* ready to drink, *A/NSB* artificial/non-nutritive sweetened beverages

**Table S4c** Median (P25-P75) daily intake (mL/day) of different fluid types and the percentage of consumers among Indonesian adults (18-65 years) by sex

|  | **18-65** **years (n=2778)** | | | |
| --- | --- | --- | --- | --- |
|  | Males (n=1256) | | Females (n=1522) | |
|  | Median (P25-P75) | % consumers | Median (P25-P75) | % consumers |
| **Total Fluid Intake** | **2553 (1822-3402)** | **100** | **2640 (1836-3515)** | **100** |
| **Water** | 1943 (1336-2700) | 100 | 2085 (1422-2880) | 100 |
| *Bottled water* | 480 (0-1886) | 65 | 479 (0-2100) | 62 |
| *Boiled tap water* | 632 (0-1769) | 56 | 576 (0-1895) | 54 |
| **Milk & derivatives** | 0 (0-0) | 25 | 0 (0-34) | 28 |
| **Hot beverages** | 240 (44-464) | 80 | 137 (0-317) | 68 |
| *Coffee* | 79 (0-305) | 63 | 0 (0-50) | 34 |
| *Tea* | 0 (0-175) | 48 | 38 (0-240) | 55 |
| **SSB** | 71 (0-303) | 60 | 86 (0-313) | 62 |
| *CSD* | 0 (0-0) | 10 | 0 (0-0) | 9 |
| *Juice-based drinks* | 0 (0-0) | 19 | 0 (0-29) | 26 |
| *Functional beverages* | 0 (0-0) | 16 | 0 (0-0) | 13 |
| *RTD Tea & Coffee* | 34 (0-214) | 52 | 29 (0-214) | 53 |
| *Flavored water* | 0 (0-0) | 9 | 0 (0-0) | 10 |
| **100% fruit juices** | 0 (0-0) | 7 | 0 (0-0) | 10 |
| **A/NSB** | 0 (0-0) | 3 | 0 (0-0) | 1 |
| **Other beverages** | 0 (0-0) | 2 | 0 (0-0) | 2 |

*SSB* sugar sweetened beverages, *CSD* carbonated sweetened drinks, *RTD* ready to drink, *A/NSB a*rtificial/non-nutritive sweetened beverages

**Table S5a** Mean daily intake (SEM) of different fluid types (mL/day) of total sample (consumers and non-consumers) of children (4-9 years) and adolescents (10-17 years) by sex

|  | 4-9 years | | | | | | 10-17 years | | | | | |
| --- | --- | --- | --- | --- | --- | --- | --- | --- | --- | --- | --- | --- |
|  | Total | | Males | | Females | | Total | | Males | | Females | |
| **TFI** | **2165** | **±45** | **2169** | **±57** | **2159** | **±71** | **2488** | **±49** | **2499** | **±65** | **2472** | **±74** |
| Water | 1663 | ±42 | 1674 | ±55 | 1644 | ±65 | 1933 | ±43 | 1941 | ±58 | 1920 | ±65 |
| *Bottled water* | *893* | *±52* | *840* | *±66* | *983* | *±85* | 1039 | ±54 | 1054 | ±68 | 1018 | ±87 |
| *Tap water* | *769* | *±48* | *833* | *±61* | *661* | *±75* | 894 | ±48 | 887 | ±65 | 903 | ±71 |
| Milk & derivatives | 254 | ±16 | 253 | ±20 | 255 | ±30 | 136 | ±11 | 123 | ±12 | 154 | ±19 |
| Hot beverages | 68 | ±9 | 78 | ±13 | 50 | ±9 | 121 | ±9 | 128 | ±11 | 112 | ±15 |
| *Coffee* | *5* | *±2* | *5* | *±2* | *6* | *±3* | 30 | ±5 | 28 | ±5 | 32 | ±11 |
| *Tea* | *62* | *±9* | *73* | *±13* | *44* | *±9* | 91 | ±7 | 100 | ±9 | 80 | ±11 |
| SSB | 167 | ±12 | 156 | ±15 | 187 | ±21 | 282 | ±16 | 296 | ±22 | 264 | ±24 |
| *CSD* | *5* | *±2* | *4* | *±2* | *6* | *±3* | 19 | ±4 | 22 | ±6 | 15 | ±4 |
| *Juice-based drinks* | *54* | *±7* | *51* | *±9* | *59* | *±9* | 56 | ±6 | 58 | ±8 | 52 | ±7 |
| *Functional beverages* | *7* | *±2* | *6* | *±2* | *9* | *±4* | 18 | ±3 | 19 | ±4 | 16 | ±4 |
| *RTD tea & coffee* | *94* | *±8* | *87* | *±9* | *107* | *±15* | 177 | ±12 | 185 | ±15 | 166 | ±20 |
| *Flavored water* | *7* | *±2* | *8* | *±3* | *7* | *±3* | 13 | ±3 | 12 | ±4 | 15 | ±5 |
| 100% fruit juices | 6 | ±2 | 3 | ±1 | 12 | ±4 | 9 | ±2 | 7 | ±2 | 11 | ±4 |
| A/NSB | 2 | ±1 | 0 | ±0 | 5 | ±2 | 3 | ±2 | 3 | ±2 | 4 | ±3 |
| Other beverages | 5 | ±2 | 5 | ±3 | 5 | ±3 | 4 | ±1 | 2 | ±1 | 7 | ±3 |

SSB Sugar sweetened beverages, CSD carbonated sweetened beverages, RTD ready to drink, A/NSD artificial/non-nutritive sweetened beverages

**Table S5b** Mean daily intake (SEM) of different fluid types (mL/day) among adults (18-65 years) by sex

|  | 18-65 years | | | | | |
| --- | --- | --- | --- | --- | --- | --- |
|  | Total | | Males | | Females | |
| **TFI** | **2721** | **±22** | **2678** | **±33** | **2756** | **±31** |
| Water | 2164 | ±20 | 2065 | ±29 | 2245 | ±29 |
| *Bottled water* | *1111* | *±25* | *1048* | *±35* | *1163* | *±35* |
| *Tap water* | *1052* | *±24* | *1017* | *±33* | *1081* | *±33* |
| Milk & derivatives | 52 | ±3 | 46 | ±4 | 56 | ±4 |
| Hot beverages | 263 | ±6 | 327 | ±10 | 210 | ±7 |
| *Coffee* | *120* | *±4* | *193* | *±7* | *59* | *±3* |
| *Tea* | *143* | *±5* | *134* | *±8* | *151* | *±6* |
| SSB | 227 | ±7 | 225 | ±10 | 229 | ±9 |
| *CSD* | *13* | *±1* | *15* | *±2* | *11* | *±1* |
| *Juice-based drinks* | *34* | *±2* | *26* | *±2* | *41* | *±3* |
| *Functional beverages* | *20* | *±2* | *21* | *±2* | *19* | *±3* |
| *RTD tea & coffee* | *148* | *±5* | *148* | *±7* | *147* | *±6* |
| *Flavored water* | *13* | *±1* | *14* | *±2* | *12* | *±1* |
| 100% fruit juices | 12 | ±1 | 10 | ±1 | 13 | ±1 |
| A/NSD | 2 | ±0 | 3 | ±1 | 1 | ±0 |
| Other beverages | 2 | ±0 | 2 | ±0 | 2 | ±0 |

*SSB* sugar sweetened beverages, *CSD* carbonated sweetened drinks, *RTD* ready to drink, *A/NSB* Artificial/non-nutritive sweetened beverages

**Table S6** Daily intake (mL/day) of total fluid intake, tap water and bottled water according to education level in the Indonesian population

|  | Mean | ±SEM | Percentiles | | | | | | |
| --- | --- | --- | --- | --- | --- | --- | --- | --- | --- |
| Education level |  |  | P5 | P10 | P25 | P50 | P75 | P90 | P95 |
|  | **TFI** | | |  |  |  |  |  |  |
| Primary school (n=531) | 2624 | ±51 | 978 | 1199 | 1721 | 2534 | 3300 | 4313 | 5106 |
| Junior high school (n=783) | 2515 | ±40 | 876 | 1201 | 1657 | 2434 | 3216 | 4005 | 4528 |
| Senior high school (n=1961) | 2664 | ±26 | 995 | 1300 | 1802 | 2549 | 3395 | 4268 | 4881 |
| Diploma / Junior college (n=132) | 2577 | ±103 | 1020 | 1203 | 1711 | 2336 | 3223 | 4413 | 5093 |
| College / University (n=237) | 2784 | ±78 | 1130 | 1309 | 1811 | 2632 | 3569 | 4526 | 5061 |

Wilcoxon signed-rank test (p<0.0001) was used to compare median of total fluid intake between education level: ^a^ significantly different from primary school,

^b^ significantly different from junior high school, ^c^ significantly different from senior high school,

^d^ significantly different from diploma/junior college, ^e^ significantly different from college/university

**Table S7** Daily intake (mL/day) of total fluid intake, tap water and bottled water according to respondent employment status in the Indonesian population

|  | Mean | ±SEM | Percentiles | | | | | | |
| --- | --- | --- | --- | --- | --- | --- | --- | --- | --- |
| Respondent employment status |  |  | P5 | P10 | P25 | P50 | P75 | P90 | P95 |
|  | **TFI** | | |  |  |  |  |  |  |
| Housewife (n=1647) | 2610 | ±28 | 991 | 1259 | 1725 | 2485 | 3310 | 4201 | 4879 |
| Not working/seeking employment (n=184) | 2724 | ±80 | 1065 | 1367 | 1739 | 2713 | 3466 | 4234 | 4674 |
| Retired (n=37) | 3009 | ±210 | 916 | 1354 | 2081 | 2980 | 3934 | 5021 | 5291 |
| Student (n=358) | 2658 | ±59 | 982 | 1406 | 1848 | 2533 | 3350 | 4207 | 4884 |
| Employed (n=1418) | 2627 | ±31 | 975 | 1200 | 1750 | 2509 | 3359 | 4311 | 4848 |
|  | **Bottled water** | | |  |  |  |  |  |  |
| Housewife (n=1647) | 1078 | ±32 | 0 | 0 | 0 | 411^d^ | 1997 | 2973 | 3600 |
| Not working/seeking employment (n=184) | 921 | ±94 | 0 | 0 | 0 | 81^d,e^ | 1694 | 3009 | 3486 |
| Retired (n=37) | 1203 | ±249 | 0 | 0 | 0 | 343 | 2743 | 3710 | 4408 |
| Student (n=358) | 1176 | ±66 | 0 | 0 | 0 | 861^a,b,e^ | 2042 | 2883 | 3600 |
| Employed (n=1418) | 1071 | ±33 | 0 | 0 | 0 | 585^b,d^ | 1886 | 2915 | 3580 |
|  | **Tap water** | |  |  |  |  |  |  |  |
| Housewife (n=1647) | 1020 | ±30 | 0 | 0 | 0 | 528^b^ | 1834 | 2811 | 3390 |
| Not working/seeking employment (n=184) | 1251 | ±98 | 0 | 0 | 0 | 965^a,d,e^ | 2282 | 3046 | 3546 |
| Retired (n=37) | 1316 | ±232 | 0 | 0 | 0 | 1100 | 2229 | 3142 | 4197 |
| Student (n=358) | 939 | ±60 | 0 | 0 | 0 | 359^b^ | 1694 | 2675 | 3111 |
| Employed (n=1418) | 954 | ±30 | 0 | 0 | 0 | 528^b^ | 1680 | 2640 | 3256 |

Wilcoxon signed-rank test (p<0.0001) was used to compare median of total fluid intake, bottled and tap water between respondent occupation: ^a^ significantly different from housewife, ^b^ significantly different from not working/seeking employment, ^c^ significantly different from retired, ^d^ significantly different from student, ^e^ significantly different from worker.

**Figure S3** Percentage of children (4-9 years), adolescents (10-17 years) and adults (18-65 years) drinking 1 serving (250 mL) of SSB daily or less per week, 2-6 servings per week and 1 serving or more per day, by sex

*M* Males, *F* Females
